# Associations of smoking and second-hand smoke exposure with obesity and obstructive sleep apnoea risk in Tuvalu

**DOI:** 10.7189/jogh.16.04250

**Published:** 2026-08-21

**Authors:** Chia-Chen Lin, Po-Jen Lin, Yu-Tien Hsu, Chia-Rui Chang, Chih-Wei Shih, Chin-Chien Kuo, Shi-Chian Shiau, Yuan-Hung Lo, Natano Elisala, Julie Elisala, Fineaso Tehulu, José Francisco López-Gil, Wen-Yi Lee, Maria Soledad Hershey, Shan-Han Chang, Jack Yu-Shuo Lu, Chien-Te Pan, Chih-Fu Wei

**Affiliations:** 1 Department of Otorhinolaryngology & Head and Neck Surgery, New Taipei Municipal Tucheng Hospital, New Taipei City, Taiwan; 2 Department of Medicine, University of Central Florida, Orlando, Florida, USA; 3 Taiwan International Cooperation and Development Fund, Taipei, Taiwan; 4 Taiwan Technical Mission to Tuvalu, Funafuti, Tuvalu; 5 Department of Social & Behavioral Sciences, Yale School of Public Health, New Haven, Connecticut, USA; 6 Center for Biostatistics in AIDS Research, Harvard T.H. Chan School of Public Health, Boston, Massachusetts, USA; 7 Ministry of Health, Tuvalu; 8 Ministry of Education and Human Resource Development, Tuvalu; 9 School of Medicine, Universidad Espíritu Santo, Samborondón, Ecuador; 10 Faculty of Health Sciences, Universidad Autónoma de Chile, Santiago, Chile; 11 Department of Epidemiology and Biostatistics, School of Public Health, Imperial College London, London, UK; 12 Human Flourishing Program at Harvard University, Institute for Quantitative Social Science, Cambridge, Massachusetts, USA; 13 Division of Gastroenterology and Hepatology, Department of Internal Medicine, National Taiwan University Hospital Yunlin Branch, Yunlin, Taiwan; 14 Institute of Medicine, Chung Shan Medical University, Taichung, Taiwan; 15 Department of Emergency Medicine, Chung Shan Medical University Hospital, Taichung, Taiwan; 16 International Medical Service Center, National Taiwan University Hospital Yunlin Branch, Yunlin, Taiwan; 17 Department of Surgery, National Taiwan University Hospital Yunlin Branch, Yunlin, Taiwan; 18 Department of Environmental and Occupational Medicine, National Taiwan University Hospital Yunlin Branch, Yunlin, Taiwan; 19 Department of Environmental and Occupational Medicine, National Taiwan University College of Medicine and Hospital, Taipei, Taiwan; 20 Department of Public Health, National Taiwan University College of Public Health, Taipei, Taiwan; 21 Institute of Epidemiology and Preventive Medicine, National Taiwan University College of Public Health, Taipei, Taiwan

**Keywords:** smoking, second-hand smoke, obesity, obstructive sleep apnoea, Pacific Islands

## Abstract

**Background:**

Tobacco use and second-hand smoke (SHS) exposure remain highly prevalent in Pacific Island nations, but evidence on their health consequences is limited. We aim to describe the prevalence of smoking and SHS exposure in Tuvalu, their associations with body measurements and sleep-related outcomes, and the heterogeneity by island of residence and age group.

**Methods:**

COMmunity-based Behaviour and Attitude surveys were conducted in Tuvalu between 2020 and 2025 among 4,066 participants from Funafuti and outer islands. Smoking status was assessed in all survey years, and SHS exposure was assessed in 2022, 2023, and 2025. Body composition (body mass index (BMI), waist and neck circumference) and obstructive sleep apnoea (OSA)-related outcomes in STOP-Bang scores were measured. We assessed the associations with multivariable linear and logistic regression, with stratified analyses by age group and island of residence.

**Results:**

Overall, 26.1% of participants were people who currently smoke and 60.7% reported SHS exposure. The mean BMI was 32.1 kg/m^2^, and 23.7% of participants had a STOP-Bang score ≥3. Current smoking (odds ratio (OR) = 1.80; 95% confidence interval (CI) = 1.30, 2.48) and SHS exposure (OR = 2.15; 95% CI = 1.54, 3.02) were associated with obesity, higher STOP-Bang scores and increased odds of STOP-Bang ≥3, which varied by island of residence.

**Conclusions:**

In Tuvalu, both active smoking and SHS exposure were associated with obesity and OSA-related outcomes, with variation by island of residence. These findings indicate the importance of strengthening the implementation and enforcement of comprehensive tobacco control for sleep health and non-communicable disease prevention in Pacific Island populations.

Tobacco smoking remains a major public health challenge in Pacific Island nations, where smoking and second-hand smoke (SHS) exposure contribute substantially to the burden of non-communicable diseases and are highly prevalent [1–6]. Previous evidence from multiple Pacific Island settings showed smoking prevalence in adults ranging from 9.3% in the Marshall Islands to 49.9% in Kiribati, and the prevalence of SHS exposure in homes and workplaces ranged from 32.4% in Tonga to 62.7% in Kiribati [2]. At the same time, marked heterogeneity exists across islands, communities, and sociodemographic groups, reflecting differences in urbanisation, household structure, employment patterns, and access to health services [2,4].

Tuvalu is a small Pacific Island nation with nine low-lying atolls, which experienced the triple threat of climate change, food insecurity, and obesity [7–9]. People in Tuvalu face multiple climate-related risks, including rising temperatures, sea-level rise, and changing rainfall patterns [8,10], which may worsen the obesity syndemic by disrupting daily life [11–13]. Our prior work showed intricate relationships between home gardening behaviours, smoking, obesity, and obstructive sleep apnoea (OSA) risk [14,15]. The allostatic load from exposure to multiple social and environmental stressors may increase cardiometabolic risk [16]; understanding these differences is important for Pacific Island populations to decrease susceptibility and mitigate the disproportionate burden of tobacco-induced diseases by promoting preventive care and tobacco control policies [6].

The adverse health effects of smoking and SHS exposure are well established, including cardiovascular disease, respiratory illness, cancer, and premature mortality [5,6,17,18]. More recently, attention has turned to less traditionally emphasised outcomes, such as obesity and OSA [18]. Impaired oxygenation, neuromuscular dysfunction, and upper airway inflammation have been proposed as shared pathophysiological mechanisms linking smoking and SHS exposure to both obesity and OSA [18–21]. Smoking has been associated with alterations in body composition and fat distribution, while SHS exposure has been linked to metabolic dysfunction, especially among children and adolescents [22–25]. Both smoking and SHS exposure increase the risk and severity of OSA across age groups; however, the underlying mechanisms and clinical implications differ between adults and adolescents [26]. In adults, tobacco use primarily acts as a synergistic catalyst that exacerbates traditional OSA risk factors, such as age-related pharyngeal narrowing and central obesity [18,26]. Conversely, in adolescents, the clinical impact of SHS exposure is often rooted in structural and developmental disruptions [21,27,28].

However, most existing evidence comes from high-income or urbanised settings, and data from Pacific Island nations remain limited [16]. In Tuvalu, where obesity prevalence is high and living environments differ substantially between the main island (Funafuti) and outer islands, the associations between tobacco smoke and SHS exposure, adiposity, and sleep health are not well characterised (**Box 1**). To address these gaps, we used data from the COMmunity-based Behavior and Attitude (COMBAT) surveys conducted in Tuvalu from 2020 to 2025. We aimed to describe the prevalence of smoking and SHS exposure among adults and adolescents, to examine their associations with body measurements and sleep-related outcomes, including OSA risk, and to explore heterogeneity by island of residence and age group.

Box 1 **Study highlights**

*What is already known on this topic* – Tobacco smoking and second-hand smoke (SHS) exposure are major contributors to non-communicable diseases worldwide. – Smoking and SHS exposure have been associated with increased risk of metabolic diseases and obstructive sleep apnoea (OSA) in studies conducted in high-income and urbanised settings. – Evidence on the health effects of tobacco exposure in Pacific Island nations is limited, despite high smoking prevalence and cardiometabolic disease burden.
*What this study adds* – In a multi-site community-based study in Tuvalu, 26.1% of participants were people who currently smoke and over 60% were exposed to SHS. – Both smoking and SHS exposure were associated with higher BMI, STOP-Bang scores and increased odds of OSA-related symptoms, including snoring, daytime fatigue, and observed apnoea. – Smoking was associated with higher body mass index and waist circumference, with stronger associations observed in the outer islands, while sleep-related associations were more pronounced in Funafuti.
*How this study might affect research, practice or policy:* – These findings extend evidence on tobacco-related health risks and highlight sleep health as an important consequence of tobacco exposure among Pacific Island populations. – Results support the need for strengthened tobacco control and SHS exposure reduction policies in Tuvalu.

## METHODS

### Study population

The COMBAT surveys were conducted in Tuvalu since 2020 as repeated cross-sectional studies to provide valuable information on sociodemographic characteristics, behavioural patterns, food consumption, garden use, and diet-related health outcomes. We included Tuvaluan participants from community and high schools using convenience sampling, and there were no exclusion criteria. Adult participants were recruited in community-based settings across different islands in Tuvalu. For the adolescents, we reached out and collaborated with the two main high schools in Tuvalu. From each school, we selected one class from forms four, five, and six (corresponding to grades 10–12) separately and administered the questionnaires to the students during their regular class activities.

During the year 2020, the first COMBAT survey was conducted at seven villages on Fongafale islet in Funafuti and two major high schools. During 2022, a second wave of the COMBAT survey was conducted across Tuvalu with around 1,000 adult participants [8]. During the year 2023, the COMBAT survey was done in Funafuti and Vaitupu to assess agricultural practices and lifestyle factors that could promote public health and environmental sustainability in Tuvalu. We incorporated the STOP-Bang questionnaire and a revised food frequency questionnaire to screen OSA since this year [15,29]. During the year 2024, we conducted household surveys to acquire semi-qualitative responses for agricultural behaviours, nutrition attitudes, and lifestyle concerns in Funafuti, and continued the school-based survey. During the year 2025, we conducted the COMBAT study across Tuvalu and included lipid profiles, blood pressure measurement, and biomarker examination.

### Exposure of interest

Smoking status was assessed in each survey year (2020, 2022, 2023, 2024, and 2025) with a single question. For the 2020 survey, we asked ‘How many cigarettes do you consume daily? (*E fia au sikaleti e pusi ite aso*?)’, and the responses were grouped as people who smoke and others. The question used in 2022, 2023, 2024, and 2025 was ‘How often do you smoke? (*Kafai koe se tino pusi, e mata e fakafia taimi e masani koe o pusi*?)’. Responses were harmonised across survey years and categorised as no smoking *vs.* others.

We assessed SHS exposure in 2022, 2023, and 2025 surveys. The exposure was assessed with a single question: ‘Do some of your family members smoke? (*E isi ne tino I loto ite otou kaiga e pusi*?)’ for these three years, and the responses were coded as binary variables (yes/no).

### Outcomes of interest

We included obesity and sleep-related health conditions that were available for multiple years. We obtained body measurements, including weight, height, and waist circumference (2020–2025), using standardised protocols [9,15], and body mass index (BMI) was calculated as weight divided by the square of height.

We assessed OSA risk using the STOP-Bang questionnaire. It should be noted that OSA-related assessments, including STOP-Bang and neck circumference, were incorporated into the study protocol beginning in 2023. Consequently, analyses involving these specific outcomes are restricted to three of the five survey waves (2023–2025). Among the subset of participants with available OSA-related data, the exact denominator for OSA-related analyses was 2,384.

The questionnaire included blood pressure, BMI, age, neck circumference, gender, and OSA-related symptoms (*i.e.* snoring, daytime fatigue, and observed apnoea) [29]. We obtained neck circumferences using standardised protocols during 2023–2025 [9,15], and hypertension was self-reported and coded as binary variables (yes/no) for the STOP-Bang score. The scores were coded as continuous variables and as binary variables with a cut-off score of ≥3.

### Covariates

We selected the covariates based on prior knowledge of their relevance to smoking and obesity and harmonised the variables into common formats for further analysis. We adjusted the models for age (in years), gender (male/female), income (above AUD 500/month for 2025 and above AUD 1,000/month for other years), and education (college and above *vs.* others). For adolescents, we adjusted the models for age and gender only. Exercise and alcohol use were additionally included in the models for sensitivity analyses.

### Statistical analysis

We leveraged the repeated cross-sectional data in COMBAT, where inputs from five different years of survey were combined as the analysis data set. We presented the data as means and standard deviations (SDs) or as numbers and percentages. We assessed group differences using analysis of variance for continuous variables and χ^2^ tests for categorical variables.

We evaluated the associations using generalised linear models and applied multivariable logistic regression to estimate the associations for binary outcomes. Analyses were conducted using complete-case analysis for each model; we excluded cases with missing values for respective outcomes or predictors. The primary sleep-health outcome was elevated OSA risk, defined as a STOP-Bang score ≥3 [29], and we also examined associations with OSA-related symptoms and adiposity indicators. We adjusted the models for age, sex, income, education, and non-communicable disease diagnoses. Stratified estimates were obtained by fitting separate models within each subgroup, such as the comparison between participants in Funafuti and other outlying islands.

We performed a sensitivity analysis to consider the residual confounding by other behavioural factors. Models were additionally adjusted for exercise and alcohol use, and we tested the associations between the outcomes of interest (*i.e.* obesity and OSA) and exercise and alcohol use. We also adjusted for survey year and excluded the data from years 2020 and 2024 to examine temporal variation across survey waves. Analyses were stratified by adults and adolescents, and models were adjusted for age and sex due to limited variability in income, education, and non-communicable disease status for adolescents. Lastly, we removed income status from the covariate list to examine potential bias from differences in income definitions across survey years.

We used *R*, version 4.4.1 (R Core Team, Vienna, Austria) for all analyses.

## RESULTS

### Descriptive statistics

We included 4,066 participants, including 3,268 adults and 798 adolescents residing in Funafuti and the outlying islands (**Table 1**). The average age was 33.0 years (SD = 15.9), and there were slightly more females (54.6%) in the study population. A large proportion (41.7%) of the study population were from Funafuti, which reflected the population composition in Tuvalu. We observed variation in income and educational attainment, with 14.2% of the study population attaining college or above degrees and 7.2% of the study population having higher monthly. As for behavioural factors, the prevalence of current smoking varied across survey years, with an overall prevalence of 26.1%. The SHS exposure remained high across 2022, 2023, and 2025, with an overall prevalence of 60.7% in the study population. Overall, 27.1% of participants reported alcohol use, 17.2% reported having a family garden, and 63.3% reported regular exercise.

**Table 1:** Demographic characteristics and health-related parameters of study participants across survey years, n (%)

	2020 (n = 550)	2022 (n = 1,132)	2023 (n = 1,100)	2024 (n = 958)	2025 (n = 326)	Total (n = 4,066)
**Demographic characteristics**						
Age, x̄ (SD)	34.6 (19.0)	39.2 (17.0)	29.6 (13.2)	30.1 (12.9)	28.2 (15.4)	33.0 (15.9)
Females	287 (53.1)	610 (53.9)	592 (54.2)	479 (52.4)	204 (67.3)	2,172 (54.6)
Funafuti residents	485 (88.2)	688 (60.8)	847 (77.0)	709 (74.4)	173 (57.5)	2,902 (71.9)
Survey sites						
*Fetuvalu High School*	115 (20.9)	52 (4.6)	109 (9.9)	94 (9.8)	27 (8.3)	397 (9.8)
*Motufoua High School*	65 (11.8)	50 (4.4)	101 (9.2)	94 (9.8)	91 (27.9)	401 (9.9)
*Communities*	370 (67.3)	1,030 (91.0)	890 (80.9)	770 (80.4)	208 (63.8)	3,268 (80.4)
Home island						
*Funafuti*	161 (29.4)	519 (45.8)	400 (36.5)	487 (53.3)	76 (30.9)	1,643 (41.7)
*Nanumea*	24 (4.4)	78 (6.9)	50 (4.6)	28 (3.1)	27 (11.0)	207 (5.3)
*Nanumaga*	56 (10.2)	52 (4.6)	55 (5.0)	18 (2.0)	41 (16.7)	222 (5.6)
*Niutao*	46 (8.4)	70 (6.2)	44 (4.0)	22 (2.4)	20 (8.1)	202 (5.1)
*Vaitupu*	68 (12.4)	99 (8.7)	225 (20.5)	111 (12.1)	31 (12.6)	534 (13.6)
*Nui*	47 (8.6)	67 (5.9)	45 (4.1)	28 (3.1)	12 (4.9)	199 (5.1)
*Nukufetau*	59 (10.8)	66 (5.8)	48 (4.4)	31 (3.4)	26 (10.6)	230 (5.8)
*Nukulaelae*	36 (6.6)	29 (2.6)	38 (3.5)	7 (0.8)	9 (3.7)	119 (3.0)
*Niulakita*	2 (0.4)	5 (0.4)	11 (1.0)	2 (0.2)	0 (0.0)	20 (0.5)
*Others*	49 (8.9)	147 (13.0)	180 (16.4)	180 (19.7)	4 (1.6)	560 (14.2)
Higher education*	163 (30.2)	160 (14.2)	260 (23.6)	65 (9.0)	89 (28.6)	737 (19.4)
Higher income*	21 (5.7)	17 (1.5)	19 (1.9)	150 (19.9)	48 (15.4)	255 (7.2)
People who smoke	83 (15.2)	354 (31.4)	284 (25.8)	27 (14.4)	110 (33.8)	858 (26.1)
SHS exposure	NA	748 (66.3)	622 (56.5)	NA	175 (55.0)	1,545 (60.7)
Alcohol use	81 (14.9)	303 (26.9)	356 (32.4)	27 (14.4)	124 (38.0)	891 (27.1)
Having family garden	NA	362 (32.0)	117 (10.6)	5 (0.6)	85 (27.1)	569 (17.2)
Exercise habit	353 (65.4)	850 (75.6)	488 (44.9)	123 (65.8)	212 (81.2)	2,026 (63.3)
**Health-related parameters**						
Height, x̄ (SD)	166.0 (9.3)	168.7 (10.4)	167.0 (9.0)	168.5 (8.2)	166.5 (10.0)	167.6 (9.4)
Weight, x̄ (SD)	87.1 (22.6)	96.3 (21.7)	88.4 (19.2)	86.9 (17.9)	84.8 (21.8)	89.8 (20.8)
BMI, x̄ (SD)	31.6 (8.2)	34.0 (8.0)	31.7 (7.1)	30.8 (7.3)	30.8 (8.4)	32.1 (7.8)
Waist circumference, x̄ (SD)	98.2 (20.0)	101.2 (16.8)	90.0 (17.2)	97.2 (14.5)	96.7 (19.0)	96.5 (17.6)
Neck circumference, x̄ (SD)	NA	NA	37.6 (4.7)	38.9 (3.6)	39.2 (7.1)	38.3 (4.8)
STOP-Bang score, x̄ (SD)†	NA	NA	1.8 (1.4)	1.3 (0.9)	2.0 (1.5)	1.8 (1.4)
STOP-Bang score ≥3†	NA	NA	262 (23.8)	15 (8.8)	90 (32.4)	367 (23.7)
Snoring	NA	NA	249 (22.8)	9 (4.8)	38 (11.8)	296 (18.5)
Daytime fatigue	NA	NA	383 (35.1)	115 (61.5)	197 (60.8)	695 (43.4)
Sleep apnoea episode	NA	NA	118 (10.8)	10 (5.3)	13 (4.0)	141 (8.8)
Having NCD	163 (30.8)	233 (20.6)	152 (13.8)	100 (11.0)	37 (11.7)	685 (17.2)
Hypertension	110 (20.6)	176 (15.6)	108 (9.8)	62 (6.8)	24 (7.5)	480 (12.0)
Diabetes	104 (19.3)	115 (10.2)	63 (5.7)	54 (5.9)	20 (6.3)	356 (8.9)
Dyslipidemia	28 (5.3)	30 (2.7)	29 (2.6)	2 (0.2)	11 (3.5)	100 (2.5)

NCD – non-communicable disease, SD – standard deviation, SHS – second-hand smoke, x̄ – mean

*Higher education is defined as education level above high school, and higher income is defined as monthly income above AUD 500/month for 2025 and above AUD 1,000/month for other years.

†STOP-Bang scores were available from 2023–2025; in 2024, the questionnaire was administered in high schools.

### Health conditions in the COMBAT population

Overall, the mean BMI was 32.1 (SD = 7.8) (**Table 1**). Mean BMI was the highest in 2022 and declined thereafter. Waist circumference showed a similar pattern, peaking in 2022 and decreasing in subsequent years of subsequent survey waves. Since 2023, OSA-related measures were assessed. The mean STOP-Bang score was 1.8 (SD = 1.4) during the three years, with 23.7% of participants having a score of ≥3. Sleep-related symptoms were common, including snoring (18.5%), daytime fatigue (43.4%), and reported sleep apnoea episodes (8.8%). Hypertension, diabetes, and dyslipidemia showed declining trends. These declines across survey waves likely reflected differences in sampling composition and survey implementation for each year.

### Regression

Because OSA-related assessments were introduced to the survey protocol in 2023, all regression models for sleep-related outcomes and neck circumference were conducted on a subset of 2,384 participants from the 2023–2025 survey waves (**Table 2**). In adjusted models, people who smoke had higher BMI (adjusted *β*-estimate = 0.94; 95% confidence interval (CI) = 0.30, 1.58; *P* = 0.004) and waist circumference than people who never smoke, but the association with neck circumference was not statistically significant. At the same time, smoking was associated with higher STOP-Bang scores and increased odds of snoring, daytime fatigue, observed apnoea episodes, and STOP-Bang score ≥3 (adjusted odds ratio (aOR) = 1.80; 95% CI = 1.30, 2.48; *P* < 0.001). We did not observe a strong association between SHS exposure and BMI, waist circumference, or neck circumference after multivariable adjustment. Meanwhile, SHS exposure was associated with higher STOP-Bang scores and increased odds of snoring, daytime fatigue, observed apnoea episodes, and STOP-Bang score ≥3 (aOR = 2.15; 95% CI = 1.54, 3.02; *P* < 0.001).

**Table 2:** Association between smoking and SHS exposure and body measurements and sleep conditions*

	Unadjusted		Adjusted†	
	**Estimate (95% CI)**	***P*-value**	**Estimate (95% CI)**	***P*-value**
**Current smoking *vs.* never smoking**				
BMI	1.13 (0.51, 1.76)	<0.001	0.94 (0.30, 1.58)	0.004
Waist circumference	3.88 (2.45, 5.32)	<0.001	2.06 (0.61, 3.51)	0.005
Neck circumference	1.66 (1.08, 2.24)	<0.001	0.18 (−0.44, 0.81)	0.564
STOP-Bang score	0.52 (0.33, 0.71)	<0.001	0.19 (0.05, 0.34)	0.009
Snoring	2.21 (1.69, 2.88)	<0.001	2.07 (1.50, 2.85)	<0.001
Daytime fatigue	1.18 (0.94, 1.47)	0.152	1.22 (0.93, 1.60)	0.142
Observed apnoea episodes	2.24 (1.50, 3.36)	<0.001	1.99 (1.32, 3.00)	0.001
STOP-Bang score ≥3	2.90 (2.26, 3.73)	<0.001	1.80 (1.30, 2.48)	<0.001
**SHS exposed *vs.* non-exposed**				
BMI	−0.23 (−0.86, 0.39)	0.459	−0.11 (−0.74, 0.51)	0.722
Waist circumference	1.76 (0.32, 3.20)	0.017	1.24 (−0.21, 2.69)	0.093
Neck circumference	0.12 (−0.43, 0.67)	0.678	0.31 (−0.28, 0.91)	0.304
STOP-Bang score	0.47 (0.28, 0.65)	<0.001	0.35 (0.21, 0.48)	<0.001
Snoring	1.63 (1.25, 2.14)	<0.001	1.60 (1.16, 2.21)	0.004
Daytime fatigue	2.07 (1.66, 2.58)	<0.001	1.90 (1.47, 2.45)	<0.001
Observed apnoea episodes	1.99 (1.39, 2.85)	<0.001	2.10 (1.34, 3.28)	0.001
STOP-Bang score ≥3	1.85 (1.44, 2.39)	<0.001	2.15 (1.54, 3.02)	<0.001

BMI – body mass index, CI – confidence interval, SHS – second-hand smoke

*Sample sizes varied by model due to the phased introduction of certain measures (*e.g.* n = 2,384 for OSA-related analyses), and neck circumference and sleep-related outcomes were assessed in the COMBAT study population since 2023.

†The adjusted model included age, gender, education attainment, income status, and non-communicable disease diagnoses as confounders.

### Stratified analysis

Associations between smoking and body measurements differed by island of residence in the adjusted models (**Table 3**). In Funafuti, smoking was not significantly associated with BMI or waist circumference. In the outer islands, smoking was associated with higher BMI (adjusted *β*-estimate = 1.33; 95% CI = 0.08, 2.59) and waist circumference. There were no significant associations with neck circumference in either setting. There was no association between SHS and BMI or neck circumference in either group. Nevertheless, SHS exposure was associated with higher waist circumference in the outer islands, but not Funafuti. For sleep-related outcomes, smoking was associated with higher STOP-Bang scores in Funafuti, but not in the outer islands. In Funafuti, smoking was associated with higher odds of snoring, observed apnoea episodes, and a STOP-Bang score ≥3. In the outer islands, smoking was associated with daytime fatigue and the odds of having a STOP-Bang score ≥3. There was a significant association between SHS exposure and higher STOP-Bang scores both in Funafuti and the outer islands. In Funafuti, SHS exposure was associated with higher odds of snoring, daytime fatigue, observed apnoea episodes, and a STOP-Bang score ≥3. In the outer islands, SHS exposure was associated with daytime fatigue and a STOP-Bang score ≥3.

**Table 3:** Stratified analysis for the association between smoking and SHS exposure on body measurements and sleep conditions, by islands of residence*

	Funafuti†		Others†	
	**Estimate (95% CI)**	***P*-value**	**Estimate (95% CI)**	***P*-value**
**Current smoking *vs.* never smoking**				
BMI	0.59 (−0.20, 1.37)	0.067	1.26 (0.09, 2.42)	0.036
Waist circumference	1.71 (−0.07, 3.50)	0.062	3.40 (0.75, 6.05)	0.012
Neck circumference	0.32 (−0.48, 1.12)	0.470	−0.20 (−1.38, 0.99)	0.746
STOP-Bang score	0.29 (0.12, 0.46)	0.082	0.30 (0.05, 0.54)	0.018
Snoring	2.18 (1.48, 3.19)	<0.001	2.12 (0.99, 4.51)	0.052
Daytime fatigue	1.26 (0.90, 1.77)	0.730	2.35 (1.30, 4.23)	0.005
Observed apnoea episodes	2.04 (1.27, 3.26)	0.007	2.26 (0.74, 6.88)	0.153
STOP-Bang score ≥3	2.07 (1.38, 3.10)	0.004	2.53 (1.16, 5.50)	0.019
**SHS exposed *vs.* non-exposed**				
BMI	−0.60 (−1.37, 0.18)	0.155	0.66 (−0.43, 1.75)	0.234
Waist circumference	0.33 (−1.47, 2.12)	0.750	2.89 (0.38, 5.41)	0.024
Neck circumference	0.44 (−0.28, 1.16)	0.229	−0.39 (−1.50, 0.73)	0.499
STOP-Bang score	0.30 (0.15, 0.45)	<0.001	0.34 (0.12, 0.56)	0.003
Snoring	1.59 (1.09, 2.31)	0.007	1.14 (0.55, 2.34)	0.728
Daytime fatigue	1.78 (1.31, 2.41)	<0.001	1.85 (1.06, 3.23)	0.030
Observed apnoea episodes	2.01 (1.22, 3.29)	0.004	2.16 (0.59, 7.87)	0.244
STOP-Bang score ≥3	2.17 (1.45, 3.26)	<0.001	2.40 (1.09, 5.29)	0.030

BMI – body mass index, CI – confidence interval, SHS – second-hand smoke

*Sample sizes varied by model due to the phased introduction of certain measures (*e.g.* n = 2,384 for OSA-related analyses), and neck circumference and sleep-related outcomes were assessed in the COMBAT study population since 2023.

†The adjusted model included age, gender, education attainment, income status, and non-communicable disease diagnoses as confounders.

### Sensitivity analysis

In the sensitivity analysis, we identified significant differences in demographic characteristics across different survey years. Additional adjustment for alcohol use and exercise status did not materially change the associations of smoking and SHS exposure with body measurements or sleep-related outcomes, and additional adjustment for survey year did not significantly alter the associations. The association between smoking and BMI was largely unchanged, whereas the association with waist circumference became slightly stronger when excluding 2020 results. The associations did not significantly change when excluding results from 2024 (Tables S1–4 in the **Online Supplementary Document**). When we examined alcohol use and exercise status as exposures, alcohol use showed limited associations with body measurements after multivariable adjustment, whereas exercise status was associated with more favourable body measurements and selected sleep-related outcomes. In age-stratified analyses, associations of smoking and SHS exposure with sleep-related outcomes were more consistent among adults than adolescents. Estimates in adolescents had wider CIs, with several associations not reaching statistical significance (Table S5 in the **Online Supplementary Document**).

## DISCUSSION

In this community-based study of 4,066 participants in Tuvalu, the prevalence of current smoking was 26.1%, and SHS exposure was 60.7%. Smoking was associated with obesity and OSA-related outcomes, including higher BMI and waist circumference, higher STOP-Bang scores, and increased odds of snoring, daytime fatigue, and observed apnoea episodes. We found SHS exposure to be associated with higher STOP-Bang scores and increased odds of multiple sleep-related symptoms. Stratified analyses showed stronger associations between smoking, SHS exposure, and sleep-related outcomes in the main island of Funafuti, while associations between smoking, SHS exposure, and body measurements were stronger on the outlying islands. Sensitivity analyses adjusting for alcohol use and exercise status, as well as the age-stratified analyses, yielded similar results. Together, these findings suggest a substantial burden of tobacco exposure and associated sleep-related risk in Tuvalu, with context-specific variation by island of residence and age group.

Our findings for smoking were consistent with prior evidence on sleep-related outcomes, and findings of SHS were consistent with prior evidence for body composition and OSA-related outcomes. For SHS, a meta-analysis showed that maternal smoking was associated with higher odds of childhood overweight (OR = 1.37) and obesity (OR = 1.45), supporting a potential role of SHS exposure on obesity [23]. Also, results from the Global School-based Student Health Survey suggested SHS exposure was associated with obesity among adolescents who reported daily SHS exposure [24], and another study showed the association between SHS exposure and abdominal obesity in Taiwan [25]. Our findings on the associations between OSA, smoking, and SHS exposure are aligned with meta-analytic evidence [18,20,21]. A systematic review and meta-analysis reported that smoking is associated with a higher apnoea-hypopnea index and lower saturation of oxyhaemoglobin, and the associations were more evident in the subgroups of severe OSA and eastern populations [18]. We found an aOR of 2.11 for OSA-SHS exposure association, which was more evident than the 35% higher risk of possible OSA among adults with SHS exposure in a previous meta-analysis [21]. This higher magnitude of association may be driven by population-specific susceptibility, including the high baseline prevalence of obesity and cardiometabolic vulnerability in Tuvalu, which could synergistically amplify the effects of SHS on upper airway inflammation. Furthermore, environmental contexts, such as prolonged exposure in multi-generational households, might result in higher cumulative SHS doses than those typically reported in other settings. However, given the cross-sectional nature of these data, this pronounced effect size should be interpreted with caution.

Meanwhile, the associations with obesity were different from earlier reports, which possibly reflected population-specific contextual factors. The positive association between smoking and BMI observed in this population contrasts with evidence from many high-income countries where people who currently smoke often have lower body weight. Prior population studies have consistently shown that people who currently smoke have lower BMI than recent quitters, likely reflecting nicotine’s effects on suppressing appetite and increasing metabolic rate [17,30]. On the other hand, studies in Japan and Taiwan have reported associations between smoking and higher BMI and computed tomography-assessed obesity indices [17,31–33]. Furthermore, another study found a U-shaped association between smoking and BMI, with higher BMI among people who smoke heavily in a Japanese worker population [34]. In Tuvalu, smoking may cluster with other obesogenic behaviours, including increased consumption of imported ultra-processed foods. For instance, individuals who can afford tobacco may also purchase more imported ultra-processed foods at stores. However, as we did not analyse dietary data, this explanation remains speculative and warrants future investigation. The potential synergy between dietary and lifestyle factors may also influence the positive association between smoking, BMI, and waist circumference we observed.

Increased airway collapsibility, intermittent hypoxaemia, sympathetic activation during sleep, and upper airway inflammation may be the underlying mechanisms for the adverse health effects of smoking and SHS exposure [35,36]. Inhaled irritants from tobacco smoke trigger chronic mucosal oedema and increase upper airway resistance, which reduces airway calibre and promotes collapsibility during sleep [37]. Recurrent sleep fragmentation and hypoxic stress further amplify sympathetic activity and systemic inflammation, contributing to insulin resistance, endothelial dysfunction, and hypertension, ultimately creating a self-perpetuating cycle [38,39]. Although nicotine initially acts as a stimulant that may increase upper airway muscle tone, nocturnal withdrawal leads to significant neuromuscular dysfunction and airway instability, further aggravating OSA [37].

Tobacco use also triggers the release of pro-inflammatory cytokines like interleukin-6 and tumour necrosis factor-alpha, which further worsens insulin resistance and increases visceral fat [40,41]. These inflammatory pathways, combined with nicotine-driven alterations in sleep architecture including prolonged sleep latency and reduced slow-wave sleep, create a metabolic milieu that reinforces the risk of both obesity and sleep-disordered breathing [42]. In high-burden settings such as Tuvalu, where obesity and metabolic disorders are prevalent, these tobacco-related sleep disturbances may further amplify population-level cardiometabolic vulnerability.

Tobacco control remains a critical public health priority in Tuvalu [43]. Although the prevalence of smoking and SHS exposure has decreased since the Tuvalu STEPS survey in 2015, both remain highly prevalent in the population [44]. Despite Tuvalu’s commitment, the 60.7% prevalence of SHS exposure we found suggests more effective implementation and enforcement of smoke-free protections [45]. There are multiple ongoing public health interventions in Tuvalu, such as smoke-free home, indoor environments, protection from SHS, and community-based cessation campaigns. These policies may help reduce smoking and SHS exposure and may yield substantial public health benefits [4,46–48]. Specifically, smoke-free legislation was associated with 10% decreased odds for all cardiovascular diseases, 17% decreased odds for respiratory system diseases, and 6% decreased odds for adverse birth outcomes [47]. Together, these results support continued investment in tobacco control and tailored interventions that account for island-specific contexts to reduce tobacco-related health burdens in Tuvalu.

Differences between Funafuti and the outer islands informed context-specific approaches for preventive smoking strategies in Tuvalu. Behavioural patterns and health conditions in Tuvalu are shaped by social, economic, and environmental contexts [16,49,50]. A previous COMBAT study showed differences in family gardening between Funafuti and the outer islands, which may contribute to variation in health outcomes [7,14]. Similarly, different smoking behaviours and SHS exposure across islands may be affected by socioeconomic status [51], workplace structure [52], community demographics [53], and social network [54]. For example, higher population density with less green space exposure may affect sleep quality among more urbanised Funafuti residents [55]. The stronger associations with body composition measures in the outer islands may reflect socioeconomic differences and access to imported foods [9]. Therefore, interventions should account for local context in Tuvalu to enhance engagement and partnership to address these public health concerns.

This study has several strengths. First, the COMBAT study is representative of a unique Pacific Island population that is underrepresented in the epidemiologic literature and addresses important public health questions for the Tuvaluan population. Second, the use of repeated community-based surveys allows assessment of temporal patterns of health conditions, demographics, and behaviours. Third, sleep-related conditions were systematically assessed using standardized and widely used instruments, which enables evaluation of both symptoms and risk indicators. Fourth, we provided information among adolescents in Tuvalu, a critical early intervention window for obesity and OSA. Although estimates among adolescents were less precise, the high prevalence of SHS exposure highlights early-life vulnerability and underscores the importance of smoke-free home policies to prevent long-term sleep and cardiometabolic consequences. Lastly, the inclusion of multiple relevant parameters and stratification analyses strengthened the interpretation of associations across subgroups and contexts.

Several limitations should be considered. First, we relied on a pooled cross-sectional design combining five distinct surveys conducted over different years. Because these surveys varied in their primary objectives, target population structures, and the specific variables collected, this heterogeneity may introduce selection bias and limit the direct comparability of data across survey waves. Consequently, the pooled data should be interpreted as an aggregate snapshot rather than a continuous cohort, and the cross-sectional nature may preclude any definitive causal inferences regarding tobacco exposure and health outcomes. For example, reverse causation cannot be excluded, although it is less plausible that OSA symptoms would increase the likelihood of smoking. Second, residual confounding by unmeasured factors could be possible. Third, biomarker data were only available in the 2025 survey. The expanded biomarker collection in future COMBAT surveys will enable validation across different years. Longitudinal studies are needed to confirm these findings and clarify underlying mechanisms. Fourth, the performance of the STOP-Bang questionnaire was not validated in Tuvaluan populations. Fifth, absolute BMI was used for adolescents instead of age- and sex-specific Z-scores due to limited region-specific reference charts; therefore, these results should be interpreted with caution. Finally, smoking and SHS exposure were assessed using a single question during the survey. Although self-reported measures can be cost-effective, the approach may introduce measurement error when examining detailed information on frequency, intensity, and duration [56,57]. We decided to use harmonised binary variables, in which the bias of misclassification would be non-differential and likely towards the null [58]. Future validation studies using the cotinine biomarker are needed to quantify the internal dose of smoking and SHS exposure.

## CONCLUSIONS

In this community-based study among adults and adolescents in Tuvalu, we presented the prevalence of smoking and SHS exposure in Tuvalu and the associations with sleep-related health outcomes. Smoking was associated with higher BMI and waist circumference. Both smoking and SHS exposure were associated with increased odds of experiencing OSA-related symptoms and higher STOP-Bang scores. These findings demonstrated the potential value of integrating comprehensive tobacco control strategies with broader cardiometabolic and sleep health interventions. Meanwhile, future longitudinal research incorporating objective, validated measurements is essential to establish causality, elucidate the underlying biological mechanisms, and inform targeted public health policies related to tobacco control.

## Additional material


Online Supplementary Document

## Data Availability

**Data availability:** Deidentified data are available upon reasonable request to the corresponding author.
