# Supplementary material for: Associations of smoking and second-hand smoke exposure with obesity and obstructive sleep apnoea risk in Tuvalu
Source: J Glob Health. 2026 Aug 21;16:04250. doi: 10.7189/jogh.16.04250 (PMC13494845; doi:10.7189/jogh.16.04250)
Supplement: Supplementary file 1 [file jogh-16-04250-s001.pdf]

Supplement to: Lin CC, Lin PJ, Hsu YT, Chang CR, Shih CW, Kuo CC, Shiau SC, Lo YH, Elisala N, Elisala J, Tehulu F, López-Gil JF, Lee WY, Hershey MS, Chang SH, Lu JYS, Pan CT, Wei CF. Associations of smoking and second-hand smoke exposure with obesity and obstructive sleep apnoea risk in Tuvalu. J Glob Health. 2026;16:04250.

Supplementary Table 1. Pairwise comparisons for descriptive characteristic differences across survey years

**Age**		**Height**		**Weight**		**Body mass index**		**Waist circumference**		**Neck circumference**		**STOP-Bang score**	
**Years**	**p-value**	**Years**	**p-value**	**Years**	**p-value**	**Years**	**p-value**	**Years**	**p-value**	**Years**	**p-value**	**Years**	**p-value**
2022-2020	<0.001	2022-2020	<0.001	2022-2020	<0.001	2022-2020	<0.001	2023-2020	<0.001	2024-2023	<0.001	2024-2023	<0.001
2023-2020	<0.001	2024-2020	<0.001	2023-2022	<0.001	2023-2022	<0.001	2023-2022	<0.001	2025-2023	<0.001	2025-2024	<0.001
2024-2020	<0.001	2023-2022	<0.001	2024-2022	<0.001	2024-2022	<0.001	2024-2022	<0.001				
2025-2020	<0.001	2025-2022	0.003	2025-2022	<0.001	2025-2022	<0.001	2025-2022	<0.001				
2023-2022	<0.001	2024-2023	0.002	2025-2023	0.049			2024-2023	<0.001				
2024-2022	<0.001	2025-2024	0.009					2025-2023	<0.001				
2025-2022	<0.001												
Study site		Gender		Hypertension		Diabetes		Dyslipidemia		Education level		Income level	
Years	p-value	Years	p-value	Years	p-value	Years	p-value	Years	p-value	Years	p-value	Years	p-value
2022-2020	<0.001	2025-2020	<0.001	2022-2020	0.019	2022-2020	<0.001	2022-2020	0.015	2022-2020	<0.001	2022-2020	<0.001
2023-2020	<0.001	2025-2022	<0.001	2023-2020	<0.001	2023-2020	<0.001	2023-2020	0.015	2023-2020	0.006	2023-2020	<0.001
2024-2020	<0.001	2025-2023	<0.001	2024-2020	<0.001	2024-2020	<0.001	2024-2020	<0.001	2024-2020	<0.001	2024-2020	<0.001
2025-2020	<0.001	2025-2024	<0.001	2025-2020	<0.001	2025-2020	<0.001	2024-2022	<0.001	2023-2022	<0.001	2025-2020	<0.001
2023-2022	<0.001			2023-2022	<0.001	2023-2022	<0.001	2024-2023	<0.001	2024-2022	<0.001	2024-2022	<0.001
2024-2022	<0.001			2024-2022	<0.001	2024-2022	0.001	2025-2024	<0.001	2025-2022	<0.001	2025-2022	<0.001
2025-2023	<0.001			2025-2022	<0.001					2024-2023	<0.001	2024-2023	<0.001
2025-2024	<0.001			2024-2023	0.025					2025-2024	<0.001	2025-2023	<0.001
Smoking		Alcohol use		SHS exposure		Home garden use		Exercise habit					
Years	p-value	Years	p-value	Years	p-value	Years	p-value	Years	p-value				
2022-2020	<0.001	2022-2020	<0.001	2023-2022	<0.001	2023-2022	<0.001	2022-2020	<0.001				
2023-2020	<0.001	2023-2020	<0.001	2025-2022	<0.001	2024-2022	<0.001	2023-2020	<0.001				
2025-2020	<0.001	2025-2020	<0.001			2024-2023	<0.001	2025-2020	<0.001				

2023-2022	0.006	2023-2022	0.006			2025-2023	<0.001	2023-2022	<0.001				
2024-2022	<0.001	2024-2022	<0.001			2025-2024	<0.001	2024-2022	0.007				
2024-2023	0.002	2025-2022	<0.001					2024-2023	<0.001				
2025-2023	0.007	2024-2023	<0.001					2025-2023	<0.001				
2025-2024	<0.001	2025-2024	<0.001					2025-2024	<0.001				
Snoring		Daytime fatigueness		Sleep apnea episode		STOP-Bang score >= 3							
Years	p-value	Years	p-value	Years	p-value	Years	p-value						
2024-2023	<0.001	2024-2023	<0.001	2024-2023	0.043	2024-2023	<0.001						
2025-2023	<0.001	2025-2023	<0.001	2025-2023	0.001	2025-2023	0.004						
2025-2024	0.013					2025-2024	<0.001						

Supplementary Table 2. Association between smoking, SHS exposure on body measurements and sleep conditions, additional adjustment for alcohol use and exercise status

	Adjusted for alcohol use				Adjusted for exercise			
	Estimate	95% CI		p-value	Estimate	95% CI		p-value
**Smokers versus non-smokers**								
BMI	1.026	0.326	1.727	0.004	0.951	0.308	1.595	0.004
Waist circumference	2.328	0.741	3.915	0.004	1.852	0.394	3.311	0.013
Neck circumference	0.120	-0.583	0.823	0.738	0.257	-0.373	0.887	0.424
STOP-Bang score	0.217	0.056	0.378	0.008	0.202	0.056	0.348	0.007
Snoring	1.998	1.396	2.858	<0.001	2.132	1.540	2.952	<0.001
Daytime fatigue	1.242	0.917	1.680	0.161	1.217	0.927	1.598	0.158
Observed apnea episodes	2.186	1.374	3.478	<0.001	2.073	1.369	3.140	<0.001
STOP-Bang score >= 3	1.834	1.272	2.644	0.001	1.864	1.344	2.585	<0.001
**SHS exposed versus non-exposed**								
BMI	-0.114	-0.751	0.522	0.725	-0.109	-0.743	0.526	0.738
Waist circumference	1.186	-0.288	2.659	0.115	1.044	-0.424	2.513	0.164
Neck circumference	0.303	-0.304	0.909	0.328	0.334	-0.274	0.942	0.281
STOP-Bang score	0.346	0.209	0.483	<0.001	0.373	0.235	0.511	<0.001
Snoring	1.532	1.105	2.124	0.010	1.655	1.191	2.301	0.003
Daytime fatigue	1.901	1.469	2.461	<0.001	2.023	1.556	2.630	<0.001
Observed apnea episodes	2.082	1.328	3.266	0.001	2.264	1.429	3.585	<0.001
STOP-Bang score >= 3	2.102	1.492	2.961	<0.001	2.173	1.541	3.064	<0.001

a. The adjusted model included age, gender, education attainment and income status as confounders.
b. Neck circumference and sleep related outcomes were assessed in COMBAT study population since 2023.

Supplementary Table 3. Association between alcohol use and exercise status on body measurements and sleep conditions

	Alcohol users versus non-smokers				People with exercise habits versus those without			
	Estimate	95% CI		p-value	Estimate	95% CI		p-value
BMI	0.208	-0.459	0.875	0.541	-0.586	-1.176	0.004	0.052
Waist circumference	0.245	-1.267	1.757	0.751	1.406	0.068	2.743	0.039
Neck circumference	0.193	-0.418	0.804	0.536	-0.593	-1.168	-0.019	0.043
STOP-Bang score	0.042	-0.098	0.182	0.557	-0.121	-0.254	0.013	0.076
Snoring	1.484	1.065	2.069	0.020	0.607	0.437	0.843	0.003
Daytime fatigue	1.067	0.819	1.389	0.631	1.107	0.864	1.419	0.422
Observed apnea episodes	1.173	0.759	1.813	0.472	0.652	0.429	0.989	0.044
STOP-Bang score >= 3	1.274	0.914	1.776	0.152	0.809	0.578	1.131	0.215

a. The adjusted model included age, gender, education attainment and income status as confounders.

b. Neck circumference and sleep related outcomes were assessed in COMBAT study population since 2023.

Supplementary Table 4. Association between smoking, SHS exposure on body measurements and sleep conditions, with additional adjustment for year of survey

	Estimate	95% CI		p-value
**Smokers versus non-smokers**				
BMI	0.773	0.137	1.410	0.017
Waist circumference	2.068	0.647	3.489	0.004
Neck circumference	0.166	-0.460	0.792	0.604
STOP-Bang score	0.249	0.117	0.380	<0.001
Snoring	2.231	1.599	3.114	<0.001
Daytime fatigue	1.016	0.778	1.326	0.908
Observed apnea episodes	2.117	1.393	3.216	<0.001
STOP-Bang score >= 3	2.025	1.429	2.871	<0.001
**SHS exposed versus non-exposed**				
BMI	-0.221	-0.846	0.404	0.489
Waist circumference	0.806	-0.616	2.227	0.267
Neck circumference	0.273	-0.322	0.868	0.369
STOP-Bang score	0.311	0.188	0.433	<0.001
Snoring	1.533	1.103	2.130	0.011
Daytime fatigue	1.932	1.484	2.514	<0.001
Observed apnea episodes	2.031	1.295	3.184	0.002
STOP-Bang score >= 3	2.133	1.493	3.048	<0.001

Supplementary Table 5. Association between smoking on body measurements and sleep conditions, excluding specific year of survey

	Excluding 2020 survey				Excluding 2024 survey			
	Estimate	95% CI		p-value	Estimate	95% CI		p-value
BMI	0.989	0.322	1.656	0.004	0.84	0.182	1.498	0.012
Waist circumference	2.717	1.194	4.241	<0.001	2.005	0.497	3.512	0.009
Neck circumference	0.314	-0.284	0.912	0.304	0.254	-0.441	0.949	0.475
STOP-Bang score	0.193	0.049	0.337	0.009	0.209	0.051	0.367	0.010
Snoring	2.068	1.503	2.847	<0.001	1.955	1.407	2.716	<0.001
Daytime fatigue	1.224	0.935	1.603	0.142	1.26	0.944	1.682	0.117
Observed apnea episodes	1.992	1.322	3.001	<0.001	2.131	1.388	3.273	<0.001
STOP-Bang score >= 3	1.796	1.301	2.478	<0.001	1.792	1.284	2.502	<0.001

Supplementary Table 6. Stratified analysis for the association between smoking, SHS exposure on body measurements and sleep conditions, by age groups

	Adults				Adolescents			
	Estimate	95% CI		p-value	Estimate	95% CI		p-value
**Smokers versus non-smokers**								
BMI	-0.125	-0.811	0.560	0.720	-0.517	-2.073	1.038	0.515
Waist circumference	0.601	-1.011	2.212	0.465	-1.993	-5.268	1.282	0.233
Neck circumference	-0.136	-0.917	0.645	0.733	0.148	-0.981	1.278	0.797
STOP-Bang score	0.162	-0.024	0.347	0.088	0.257	0.027	0.487	0.029
Snoring	1.542	1.100	2.162	0.012	2.546	0.718	9.027	0.148
Daytime fatigue	1.251	0.911	1.718	0.166	1.942	1.039	3.631	0.038
Observed apnea episodes	1.826	1.156	2.884	0.010	1.600	0.495	5.173	0.433
STOP-Bang score >= 3	1.544	1.087	2.193	0.015	2.436	0.949	6.257	0.064
**SHS exposed versus non-exposed**								
BMI	-0.524	-1.205	0.158	0.132	0.622	-0.594	1.837	0.317
Waist circumference	0.621	-1.004	2.245	0.454	1.302	-1.279	3.884	0.323
Neck circumference	0.215	-0.508	0.938	0.560	0.154	-0.851	1.158	0.764
STOP-Bang score	0.342	0.171	0.513	<0.001	0.221	0.023	0.419	0.030
Snoring	1.400	1.002	1.955	0.048	8.597	0.958	77.151	0.055
Daytime fatigue	2.071	1.524	2.814	<0.001	1.609	0.988	2.621	0.056
Observed apnea episodes	1.823	1.120	2.967	0.016	2.896	0.893	9.388	0.076
STOP-Bang score >= 3	1.997	1.401	2.848	<0.001	4.557	1.299	15.983	0.018

Supplementary Table 7. Association between smoking, SHS exposure on body measurements and sleep conditions after removing income from confounder

	Estimate	95% CI		p-value
**Smokers versus non-smokers**				
BMI	0.960	0.351	1.569	0.002
Waist circumference	2.411	1.019	3.803	<0.001
Neck circumference	0.443	-0.167	1.052	0.155
STOP-Bang score	0.164	0.027	0.302	0.019
Snoring	1.851	1.373	2.494	<0.001
Daytime fatigue	1.273	0.983	1.647	0.067
Observed apnea episodes	1.849	1.249	2.738	0.002
STOP-Bang score >= 3	1.633	1.203	2.216	0.002
**SHS exposed versus non-exposed**				
BMI	-0.181	-0.786	0.424	0.558
Waist circumference	1.321	-0.095	2.737	0.068
Neck circumference	0.270	-0.284	0.824	0.34
STOP-Bang score	0.343	0.215	0.471	<0.001
Snoring	1.523	1.134	2.047	0.005
Daytime fatigue	1.994	1.563	2.543	<0.001
Observed apnea episodes	2.049	1.345	3.120	<0.001
STOP-Bang score >= 3	2.053	1.498	2.814	<0.001
